# Catecholaminergic polymorphic ventricular tachycardia with diagnostic difficulty differentiating seizures from syncope: a case report

**DOI:** 10.3389/fped.2026.1950509

**Published:** 2026-09-18

**Authors:** Takato Akiba, Shino Shimada, Kantaro Kimura, Yosuke Baba, Hiromichi Shoji, Jun Yoshimoto

**Affiliations:** 1 Department of Pediatrics, Juntendo University Shizuoka Hospital, Shizuoka, Japan; 2 Department of Pediatrics and Adolescent Medicine, Juntendo University Graduate School of Medicine, Tokyo, Japan; 3 National Epilepsy Center, NHO Shizuoka Institute of Epilepsy and Neurological Disorders, Shizuoka, Japan; 4 Medical Genomics Center, National Institute of Global Health and Medicine, Tokyo, Japan; 5 Department of Arrhythmia Medicine, Shizuoka Children's Hospital, Shizuoka, Japan

**Keywords:** catecholaminergic polymorphic ventricular tachycardia, epilepsy mimics, exercise testing, misdiagnosis, semiology of pediatric syncope

## Abstract

**Introduction:**

Catecholaminergic polymorphic ventricular tachycardia (CPVT) is a potentially fatal inherited arrhythmia syndrome triggered by exercise or emotional stress that may mimic epileptic seizures, resulting in delayed diagnosis.

**Case presentation:**

We report the case of a 6-year-old girl with recurrent transient loss of consciousness (T-LOC) who was initially misdiagnosed with epilepsy. She experienced three episodes of T-LOC between 4 and 6 years of age. The first two episodes occurred during or immediately post-exercise, whereas the third appeared non-exertional; however, retrospective review suggested fear-induced sympathetic activation as the trigger. During the second and third episodes, she exhibited eye opening, fixed gaze, and tonic posturing, closely resembling epileptic seizures. Interictal electroencephalography was normal, resting electrocardiography (ECG) showed sinus bradycardia without QT prolongation, and an initial Master's triple two-step exercise test increased the heart rate to only 74 beats per minute (bpm) without inducing arrhythmia. She was provisionally diagnosed with epilepsy, and a diagnostic trial of levetiracetam was initiated. Four months later, school-based ECG screening, prompted by her history of seizure-like episodes with impaired consciousness, led to further cardiac evaluation, which revealed bigeminal premature ventricular contractions. Subsequent treadmill exercise testing at a tertiary center induced bidirectional ventricular tachycardia at a heart rate of 144 bpm, confirming the diagnosis of CPVT. Genetic testing identified a pathogenic ryanodine receptor 2 (*RYR2)* variant (c.11836G > A, p.Gly3946Ser). Treatment with nadolol and flecainide was initiated, levetiracetam was discontinued, and she has remained free of syncope and seizure-like episodes for >2 years.

**Conclusion:**

This case highlights the diagnostic challenges of CPVT, and underscores the need for specialized evaluation, including repeat exercise testing under varying conditions, in children with exercise- or emotion-induced T-LOC suggestive of cardiac syncope.

## 1 Introduction

Transient loss of consciousness (T-LOC) can be broadly classified into syncope, which results from transient global cerebral hypoperfusion, and nonsyncopal causes of impaired consciousness, such as epileptic seizures (1). T-LOC is a relatively common presentation in pediatric practice, most often resulting from benign conditions such as vasovagal syncope. However, cardiac syncope is an important differential diagnosis because it may be associated with life-threatening arrhythmias and sudden cardiac death, making early diagnosis and appropriate intervention critical for improving patient outcomes (2).

Pathogenic ryanodine receptor 2 (RYR2) variants are known to cause three distinct types of disease: catecholaminergic polymorphic ventricular tachycardia (VT) (CPVT), catecholaminergic release deficiency syndrome (CRDS), and exon 3 deletion syndrome (E3DS) (3–5). CPVT is an inherited arrhythmia syndrome most commonly caused by pathogenic variants in the *RYR2* gene. It is characterized by exercise- or emotion-induced polymorphic or bidirectional VT (Bi-VT), which can result in syncope or sudden cardiac death. Patients with CPVT typically have normal resting electrocardiographic (ECG) findings, and their clinical presentation may lead to an initial misdiagnosis of epilepsy or vasovagal syncope (6). These conditions are primarily differentiated through careful history-taking; however, determining the underlying cause can be challenging when witness accounts are unavailable or when the clinical presentation is atypical. Although exercise testing is central to the diagnosis of CPVT, the sensitivity of a single exercise test is limited and may depend on the exercise modality and the achieved heart rate. Consequently, CPVT may remain undiagnosed despite apparently normal initial investigations (3, 7).

Herein, we report the case of a child with CPVT caused by a pathogenic *RYR2* variant. Her seizure-like manifestations strongly suggested epilepsy, whereas her exercise-induced T-LOC raised suspicion of cardiac syncope. Establishing the diagnosis required multiple exercise provocation tests to elicit the characteristic arrhythmia, ultimately leading to a definitive diagnosis.

## 2 Case presentation

A 6-year-old girl presented with recurrent T-LOC beginning at 4 years of age. Her perinatal and developmental history was unremarkable. Her family history was notable for adult-onset arrhythmias in her father and maternal great-aunt, although the specific diagnoses remained unknown despite detailed history-taking. There was no family history of sudden death at a young age.

At 6 years of age, while attending kindergarten during a traditional Japanese event, she watched a bean-throwing demonstration featuring *Oni* (a traditional Japanese demon-like character). She gradually backed away in apparent fear before falling backward and striking the occipital region of her head against a plastic table. During the fall, her eyes remained open with a fixed gaze, she was unresponsive to verbal stimuli, and tonic posturing of the extremities was observed, without automatisms, tongue biting, or urinary incontinence. The episode lasted approximately 4 min and was followed by postictal sleepiness and transient speech disturbance, without postictal confusion, both of which resolved spontaneously within 2 h. Physical and neurological examinations were unremarkable, and postictal blood tests revealed no abnormalities.

She was referred to our neurology outpatient clinic, where a detailed history was obtained for each of the T-LOC episodes that had occurred at 4 and 5 years of age, including the onset, associated symptoms, duration, and recovery course. At 4 years of age, she suddenly fell forward during exercise with her eyes closed and became unresponsive. The episode lasted approximately 40 s and was associated with urinary incontinence but no convulsive movements,automatisms, or tongue biting, with rapid recovery of consciousness and without postictal confusion. At 5 years of age, she fell forward immediately after exercise at kindergarten, sustaining a mandibular injury. Following the fall, she opened her eyes with a fixed gaze and exhibited predominantly tonic posturing of the extremities for approximately 40 s, without automatisms, tongue biting, or urinary incontinence, before rapidly regaining consciousness without postictal confusion. Interictal electroencephalography (EEG) performed at 6 years of age demonstrated a normal background without epileptiform discharges.

Based on these clinical features, which were inconsistent with vasovagal syncope, together with the exertional nature of the T-LOC episodes, cardiac syncope was suspected, prompting further evaluation. A resting 12-lead electrocardiogram (ECG) demonstrated sinus bradycardia with a heart rate of 53 beats/min without QT prolongation (QTc, 381 ms by Bazett's formula), ST-T abnormalities, or conduction disturbances (Figure 1). Echocardiography revealed no structural cardiac abnormalities. An exercise ECG was performed using the Master's triple two-step exercise test, during which she ascended and descended a two-step staircase for 4 min and 30 s. Sinus rhythm was maintained throughout the test, with no QT prolongation and only isolated premature atrial contractions observed (Figure 2). The maximum heart rate achieved was only 74 beats/min, which was considered insufficient to provide adequate adrenergic stress for reliable arrhythmia provocation.

**Figure 1:**
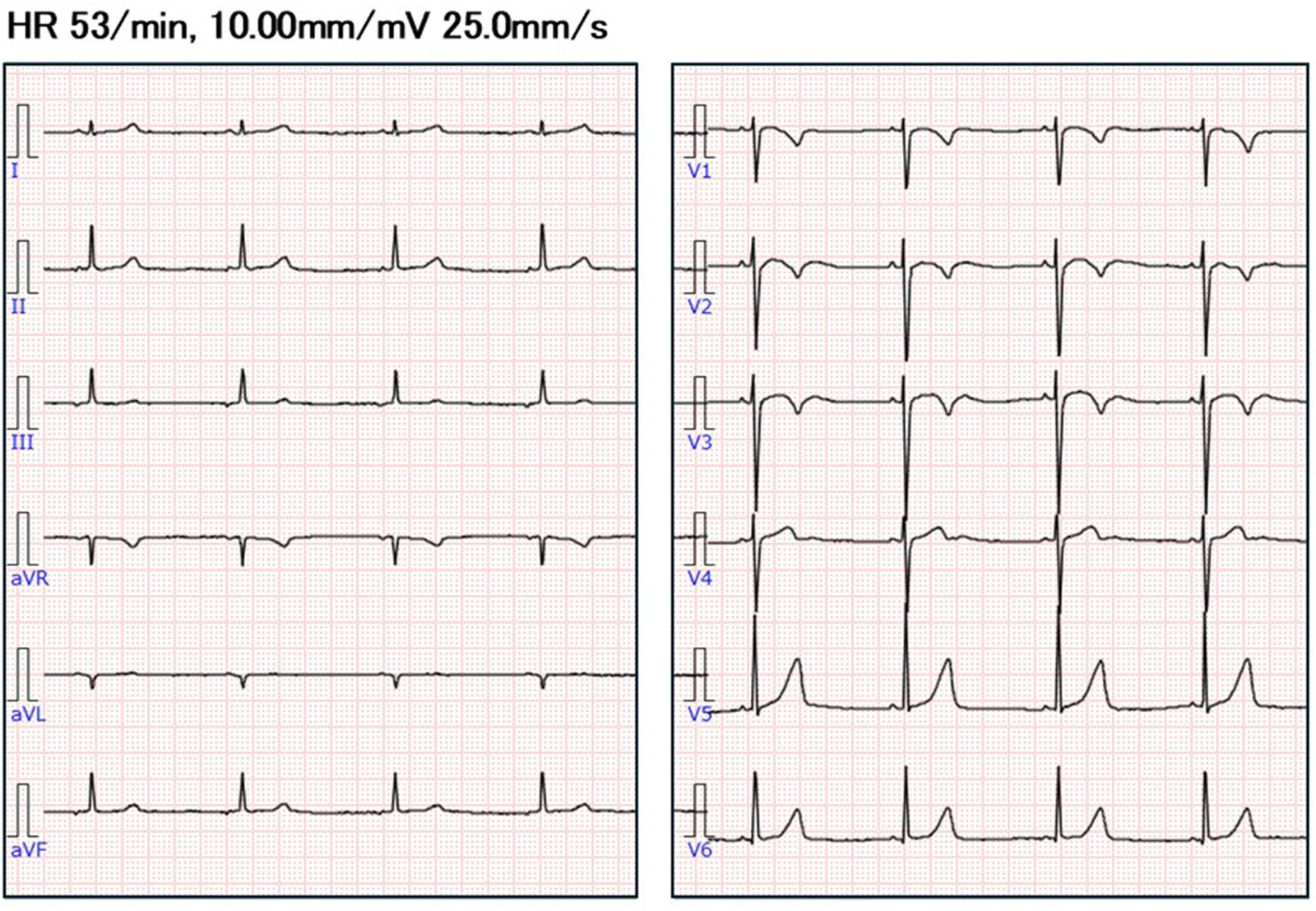
Resting 12-lead electrocardiogram at the initial visit. The resting electrocardiogram showed sinus rhythm without QT prolongation.

**Figure 2:**
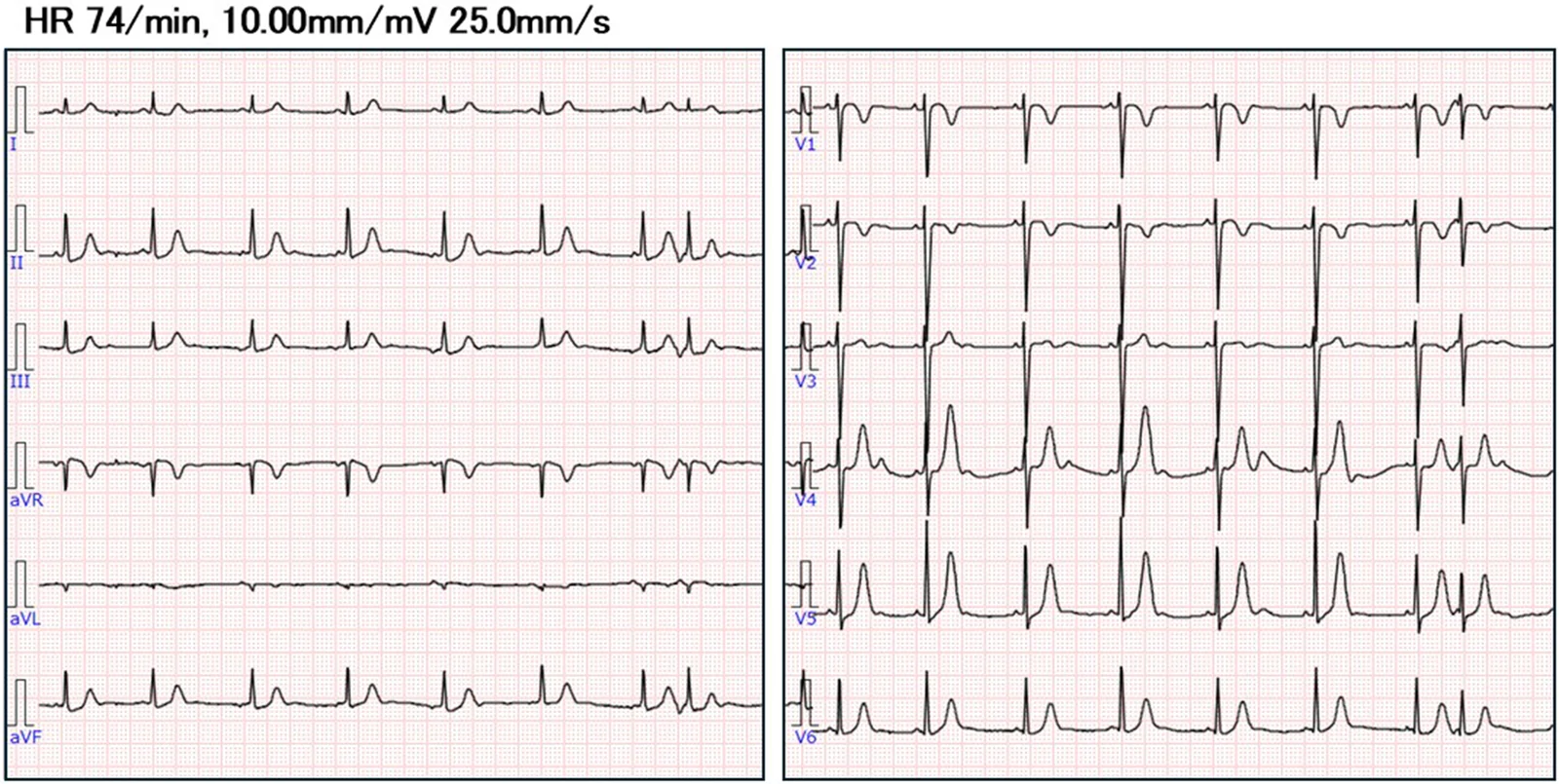
12-lead electrocardiogram after a master's triple two-step exercise test at the initial visit. At the initial hospital visit, the treadmill exercise test was considered difficult because of the patient's young age. Instead, a Master's triple two-step exercise test was conducted in which the patient ascended and descended a two-step staircase for 4 min and 30 s, respectively. No QT interval prolongation was observed; only isolated premature atrial contractions were detected.

Her clinical findings were subsequently reassessed (Table 1), and the characteristics of each T-LOC episode were carefully reviewed. The initial episode occurred during exercise and was considered suggestive of cardiac syncope. In contrast, the second episode, although occurring immediately after exercise, exhibited seizure-like features, including eye opening and a fixed gaze. The third episode lacked overt cardiac features and appeared more consistent with an epileptic seizure. No objectively observable features suggestive of vasovagal syncope, such as dizziness, nausea, or diaphoresis, were reported. In the absence of inducible arrhythmias on exercise testing, a definitive diagnosis of cardiac syncope could not be established. Taken together, because objective evidence supporting a cardiac etiology was lacking, epilepsy was considered the most likely working diagnosis at that time, and levetiracetam (10 mg/kg/day) was initiated as a diagnostic therapeutic trial.

**Table 1:** Clinical features of the three transient loss of consciousness (T-LOC) episodes and family history.

Episode	Findings suggestive of cardiac syncope	Findings suggestive of seizure
1st T-LOC	During exercise, eyes closed, rapid recovery of consciousness, short duration(<1 min)	None
2nd T-LOC	During exercise, rapid recovery of consciousness, short duration(<1 min)	Eyes open, fixed gaze
3rd T-LOC	Trigger present: fear of a Setsubun demon during bean-throwing event (No exercise-induced episodes)	Eyes open, fixed gaze, prolonged duration (∼4 min), transient speech disturbance
Family history	Positive for arrhythmia (father and maternal great-aunt)	None

Fortunately, no further episodes of T-LOC occurred. Four months later, she underwent routine school-based ECG screening. Although the resting ECG showed no abnormalities, she was referred for secondary evaluation because of her history of seizure-like episodes with impaired consciousness. During the secondary evaluation, a Master's double two-step exercise test (3 min of stair climbing) revealed bigeminal premature ventricular contractions (Bi-PVCs). She was subsequently referred to a tertiary care center for comprehensive cardiac assessment. Repeat Master's double two-step testing and treadmill exercise testing induced Bi-VT, establishing the diagnosis of CPVT (Figure 3). Catecholamine provocation testing was therefore not required. Subsequent genetic testing identified a previously reported pathogenic missense variant in *RYR2* (c.11836G > A, p.Gly3946Ser) located in exon 88, a recognized hotspot for CPVT-causing *RYR2* variants. As genetic testing was performed only in the proband, whether this variant is *de novo* or inherited remains undetermined, and cascade cardiac and genetic screening of her parents has not yet been performed. Treatment with nadolol and flecainide was initiated, levetiracetam was discontinued, and she has remained free of syncope and seizure-like episodes for >2 years of follow-up.

**Figure 3:**
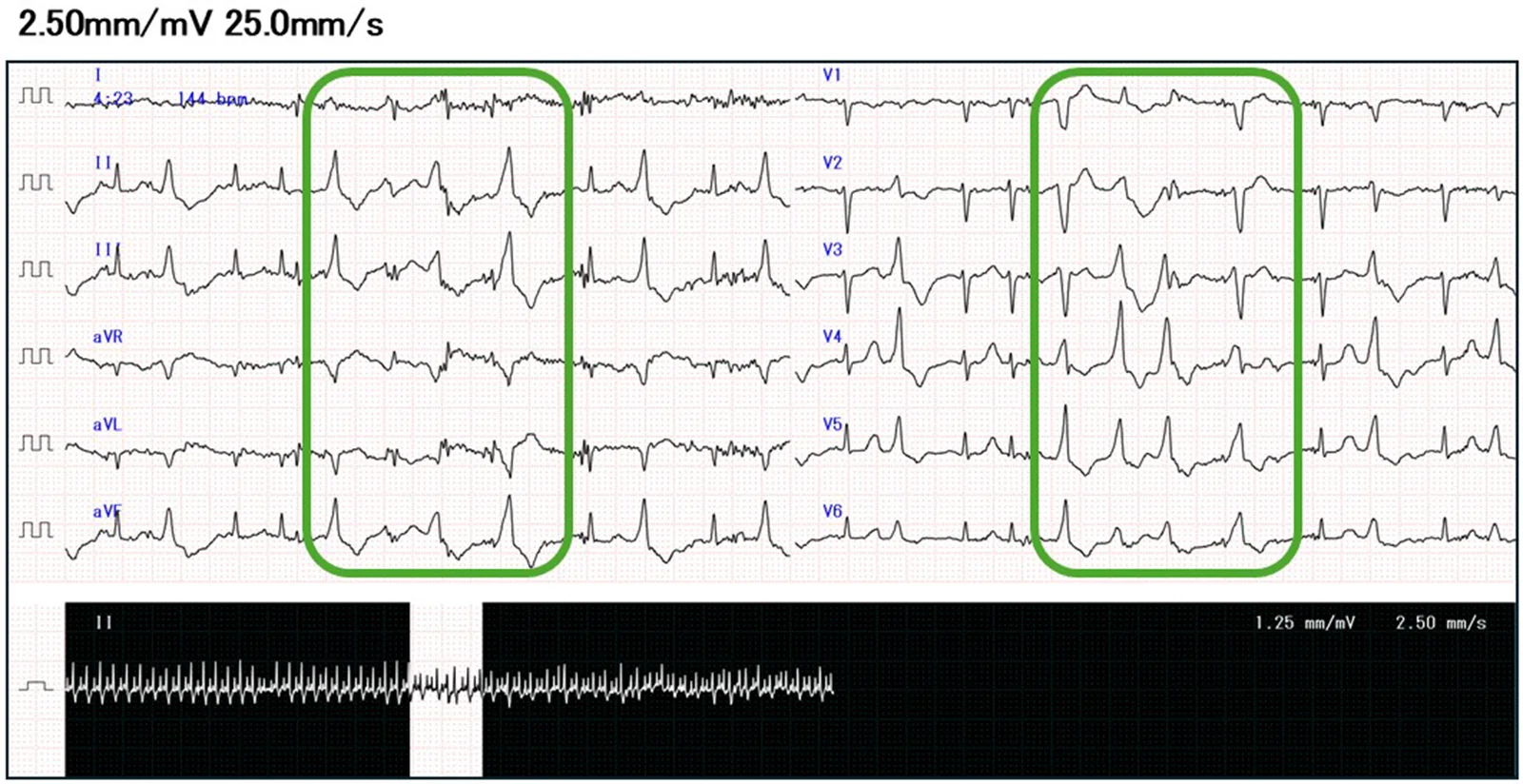
Results of a treadmill exercise test conducted at a tertiary care center. A treadmill exercise test was subsequently conducted at a tertiary care center based on the results of Master's double two-step exercise test. Bidirectional ventricular tachycardia was induced 1 min after exercise.

## 3 Discussion

This report describes a pediatric patient with CPVT who experienced recurrent T-LOC associated with exercise and exhibited features suggestive of both epileptic seizures and cardiac syncope. Because CPVT can result in syncope or sudden cardiac death, careful differentiation from epileptic seizures is essential. CPVT typically presents during childhood, adolescence, or young adulthood, has an estimated prevalence of approximately one in 10,000, and is associated with an untreated 10-year mortality rate of approximately 40% (8).

In our patient, a normal resting ECG and initial exercise testing that failed to provoke arrhythmia led to a provisional diagnosis of epilepsy. School-based ECG screening subsequently prompted further cardiac evaluation, based solely on her history of seizure-like episodes rather than any ECG abnormality. Subsequent exercise testing revealed Bi-PVCs, leading to referral to a tertiary center, where CPVT was confirmed. This case highlights several key diagnostic challenges: ECG abnormalities in CPVT may be difficult to provoke, T-LOC may be triggered by both exercise and emotion, and clinical information obtained during events is often limited. These factors complicate the differentiation between epilepsy and cardiac syncope in children.

Because resting ECG and echocardiographic findings are commonly normal, diagnosis requires induction of polymorphic VT or Bi-VT, typically by exercise ECG. Although Bi-VT on exercise ECG is highly specific, with a reported specificity of approximately 97%, its sensitivity is only approximately 50%, creating a potential diagnostic bottleneck (7). The typical heart-rate threshold for detecting premature ventricular contractions or VT has been reported to range from 110 to 150 beats/min (3). In our patient, the initial Master's triple two-step exercise test achieved a maximum heart rate of only 74 beats/min, which was insufficient to provoke Bi-VT. In contrast, Bi-VT occurred at 144 beats/min during treadmill testing and at 162 beats/min during a repeat Master's double two-step test at the tertiary center.

As CPVT-related arrhythmias may resolve rapidly after exercise, tests that primarily capture post-exercise changes, such as the Master's two-step test, may fail to detect them. Treadmill testing is therefore preferable, although it may be unavailable for young children in many Japanese hospitals (3, 9). Because arrhythmia provocation carries a risk of inducing VF, early evaluation at a specialized pediatric arrhythmia center is essential when cardiac syncope cannot be excluded.

CPVT can induce life-threatening arrhythmias not only during exercise, but also in response to emotional stress, including psychological tension, startle, or fear (10). In the present case, the first and second T-LOC episodes occurred during or immediately after exercise, consistent with the typical exertion-related presentation of CPVT. In contrast, the third episode had no obvious exertional trigger, although the group activity witnessed immediately beforehand may have elicited a fear response that triggered the episode via increased sympathetic activity. Although the intensity of the stimulus was not documented in detail, a pediatric case of CPVT resulting in cardiac arrest during an amusement park ride has previously been reported (11). Particularly in children, relatively minor emotional stimuli encountered in daily life may precipitate symptoms. Therefore, when obtaining a history of the circumstances immediately preceding T-LOC, clinicians should assess not only exertional but also emotional triggers.

In this case, eye opening and a fixed gaze during the second and third T-LOC episodes initially supported a diagnosis of epileptic seizures. Given the rarity of her episodes, capturing an ictal EEG recording was not feasible, and the interictal EEG obtained does not reliably exclude epilepsy. Although coincidental unprovoked epileptic seizures cannot be completely excluded, the final diagnosis of CPVT makes a nonepileptic interpretation more plausible. Eye closure is generally considered a useful feature for distinguishing epileptic from nonepileptic events (12), but eye opening and upward deviation have also been reported during syncope, and the duration of deviation (brief in syncope, sustained in seizures) may be more informative than the presence or absence of eye opening alone (13).

Although witness recall of features such as ocular deviation, muscle tone, and seizure duration can be reasonably accurate (14), witnesses are often family members observing events under emotional distress, and individual clinical features should therefore not be overinterpreted. Instead, a comprehensive assessment integrating precipitating triggers, the complete clinical semiology, and the overall context is essential, given the potential for misinterpretation.

## 4 Conclusion

Overall, this pediatric case illustrates that T-LOC may be triggered by both exercise and emotional stress in patients with CPVT. The clinical presentation closely mimicked epileptic seizures, leading to an initial misdiagnosis of epilepsy. This case also highlights that a single submaximal exercise test may fail to detect CPVT-associated arrhythmias, as successful arrhythmia induction depends on both the exercise modality and the achieved heart rate. Therefore, a negative initial exercise test does not definitively exclude the diagnosis. Moreover, semiological features such as eye opening or ocular deviation are not specific to epileptic seizures and may also occur in cardiac syncope, limiting their diagnostic value when interpreted in isolation. In pediatric patients with recurrent T-LOC associated with exercise or emotional stress, CPVT should be considered even when initial investigations are unremarkable, and careful reassessment, including repeat exercise testing under appropriate conditions, may be essential for establishing an accurate diagnosis.

## Data Availability

The datasets presented in this article are not readily available because of ethical and privacy restrictions. Requests to access the datasets should be directed to the corresponding author.
